# Laryngeal Adenoid Cystic Carcinoma—Two Case Reports and Literature Review

**DOI:** 10.1155/crip/4744129

**Published:** 2026-04-08

**Authors:** Katarina Jovanovic, Sasa Jakovljevic, Neda Mladenovic, Nemanja Radivojevic, Djurdjina Kablar, Ana Marija Tomic, Hristina Glogovac, Zoran Dudvarski

**Affiliations:** ^1^ Clinic of Otorhinolaryngology and Maxillofacial Surgery, University Clinical Center of Serbia, Belgrade, Serbia, kcs.ac.rs; ^2^ University of Belgrade-Faculty of Medicine, Belgrade, Serbia, bg.ac.rs; ^3^ Department of Pathology, Histopathology and Medical Cytology, University Clinical Center of Serbia, Belgrade, Serbia, kcs.ac.rs; ^4^ Institute of Pathology, Faculty of Medicine, University of Belgrade, Belgrade, Serbia, bg.ac.rs

**Keywords:** adenoid cystic carcinoma, larynx, metastasis, surgery

## Abstract

Laryngeal adenoid cystic carcinoma (LACC) is a rare malignant tumor, representing less than 1% of all laryngeal cancers. This tumor arises from the minor salivary or mucous glands of the upper respiratory tract and is characterized by slow growth, local invasiveness, and a high propensity for distant metastases. The submucosal extension and lack of symptoms often delay diagnosis. Such features present notable challenges in clinical practice.We report two cases of LACC in elderly male patients with clinically advanced laryngeal tumors. The first case involved a 73‐year‐old patient with bilateral laryngeal paralysis and subglottic stenosis caused by a tumor. Despite a prior history of tracheal adenoid cystic carcinoma treated 9 years earlier, recurrence was considered unlikely based on the available clinical, radiological, and pathological findings. Histopathological analysis confirmed the diagnosis of LACC. The patient underwent total laryngectomy, bilateral selective neck dissection, and right hemithyroidectomy. Postoperative histopathology classified the tumor as pT4aN0, and the patient remains recurrence‐free 24 months post‐surgery.The second case involved a 79‐year‐old patient presenting with persistent hoarseness and right‐sided laryngeal immobility. Computed tomography scan of the neck revealed a hyperdense paraglottic mass causing laryngeal stenosis and thyroid cartilage erosion. After histopathological confirmation of LACC on an open laryngeal biopsy specimen obtained during laryngofissure, the patient underwent total laryngectomy with selective neck dissection and received postoperative radiotherapy at a total dose of 63 Gy in 30 fractions. Seventeen months after treatment, the patient remains in good health without recurrence.These two cases of LACC, both presenting in elderly male patients without traditional risk factors, showed advanced transglottic extension with cartilage invasion and differing histological patterns—cribriform/tubular in one case and predominantly solid in the other. Despite the challenges in achieving early diagnosis due to submucosal tumor growth, both patients responded favorably to surgery with or without adjuvant radiotherapy. The observed variability in macroscopic and microscopic features underscores the need for individualized treatment strategies and supports the potential value of detailed histopathological classification in guiding therapeutic decision‐making for this rare tumor type.

## 1. Introduction

Laryngeal cancer is the second most common malignancy in the head and neck region and accounts for 1.1% of all malignant diseases [1]. Laryngeal adenoid cystic carcinoma (LACC) was first described in 1853 by Robin and Laboulbene and was previously named cylindroma [2]. This type of cancer is considered one of the rarest types of all laryngeal malignancies (< 1%) [1]. This tumor arises from the minor salivary or mucous glands of the upper respiratory tract [3]. The etiopathogenesis is not fully elucidated and is presumed to be multifactorial [3]. This tumor type is characterized by slow growth, extensive local invasiveness, frequent recurrence, and late‐onset distant metastases, necessitating prolonged postoperative surveillance [4]. In this report, we present two rare cases of LACC in elderly male patients, both with advanced local extension and distinct histopathological growth patterns, underscoring the diagnostic difficulties and therapeutic challenges of this uncommon malignancy. A brief review of the literature is also provided to contextualize these findings.

## 2. Case Presentation

### 2.1. Case No. 1

A 73‐year‐old man presented with a 5‐month history of persistent cough, fatigue, and progressive dysphagia. Indirect laryngoscopy showed bilateral vocal fold paralysis and mucosal irregularity suspicious for neoplasia, with marked narrowing of the glottic airway. No palpable cervical lymphadenopathy was present on physical examination. Contrast‐enhanced computed tomography of the neck revealed a right‐sided subglottic mass (Figure 1a) without evidence of regional lymph node enlargement. Additional imaging procedures, including chest CT scan and abdominal ultrasound, showed no evidence of distant metastatic disease at that time. The patient’s medical history was notable for adenoid cystic carcinoma (ACC) of the trachea diagnosed 9 years earlier (Figure 1b). He initially received systemic chemotherapy with gemcitabine/cisplatin, a regimen commonly used in palliative treatment of salivary gland–type carcinomas. Subsequent radiological follow‐up demonstrated multiple hypodense lesions in the liver and adrenal glands, the largest measuring approximately 2.3 cm, which were considered radiologically suspicious for metastases. This assessment was based on radiologic features reported in the literature and the known metastatic potential of ACC; however, histopathological confirmation was not obtained [5]. The patient subsequently received six additional cycles of the same chemotherapy regimen, followed by maintenance gemcitabine. On serial CT imaging (neck, chest, abdomen, and pelvis), the previously identified liver and adrenal lesions were no longer detected on follow‐up CT imaging. Minimal residual hypodense hepatic changes without radiological features of malignancy persisted and were interpreted as nonspecific and non‐progressive. Two years later, disease recurrence was treated with external beam radiotherapy (44 Gy in 22 fractions), resulting in complete radiological remission on follow‐up CT imaging.

Figure 1Case no. 1: CT, intraoperative and endoscopic findings of laryngeal tumor. (a) An axial CT scan of the neck revealed an infiltrative subglottic tumor mass causing laryngeal stenosis (arrow). (b) Endoscopic view of the trachea demonstrated adenoid cystic carcinoma. The lesion was located in the proximal trachea and showed mucosal irregularity with partial obstruction. (c) The lesion measured approximately 44 × 30 × 15 mm and caused subglottic narrowing (arrow).

**Figure:**
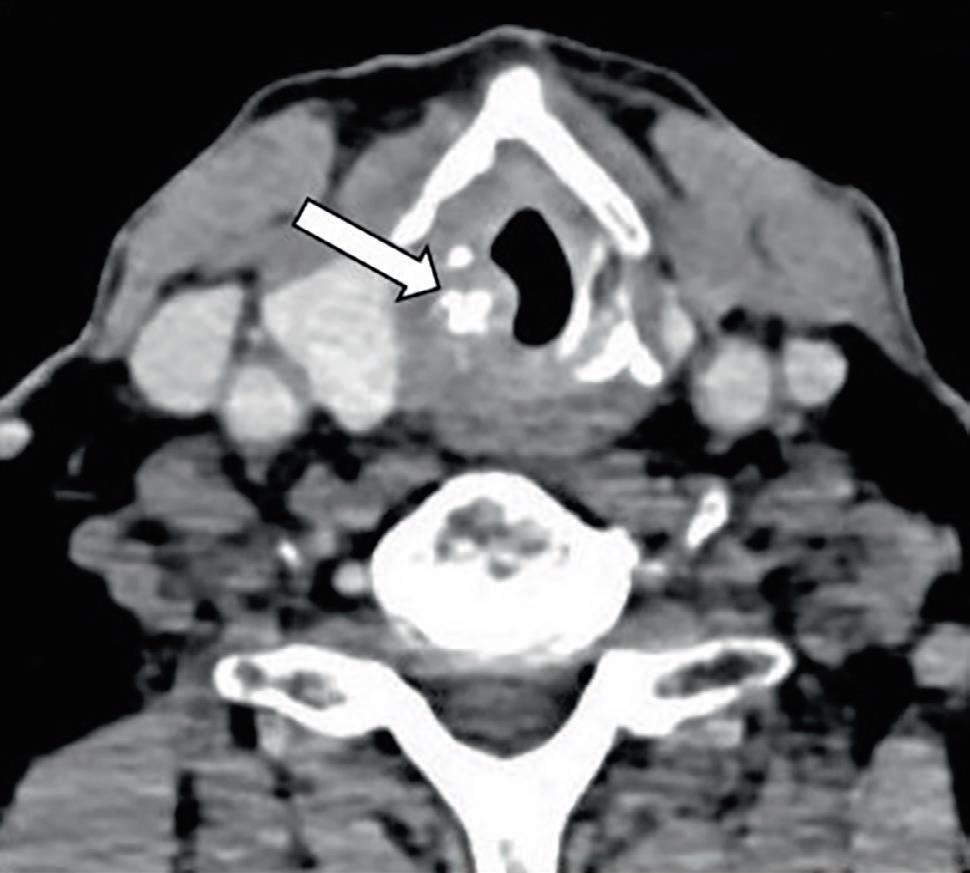
(a)

**Figure:**
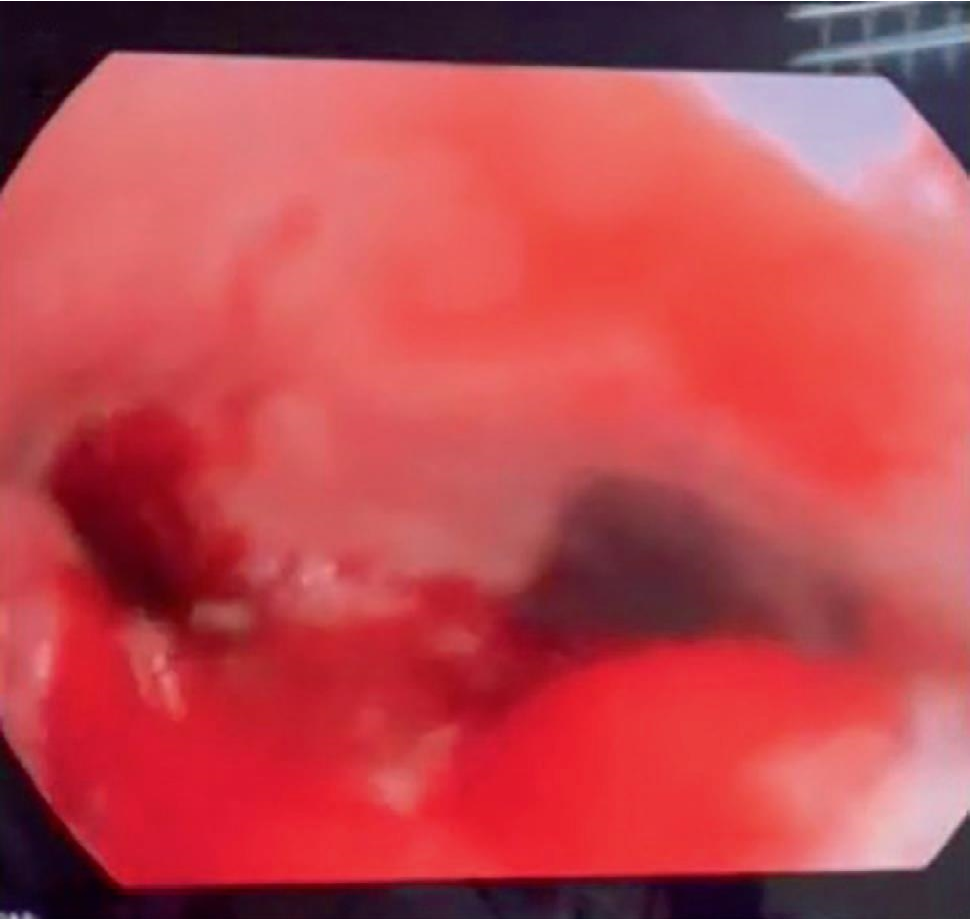
(b)

**Figure:**
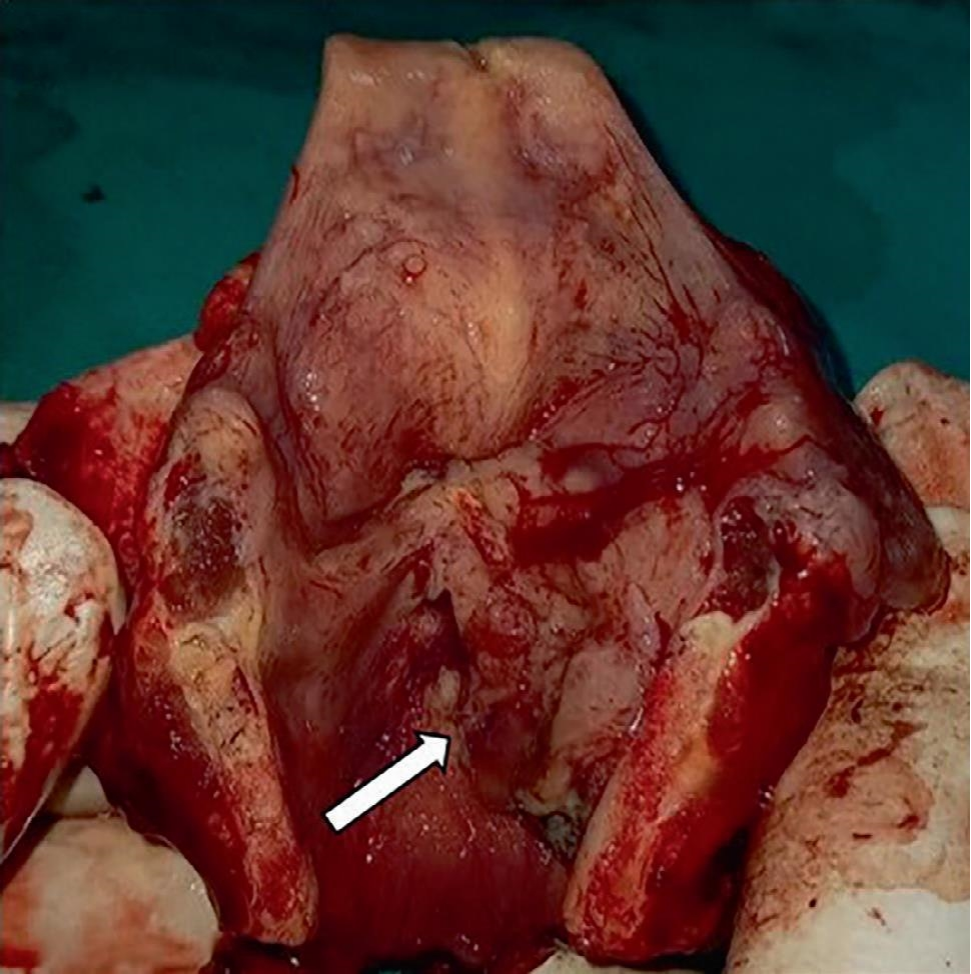
(c)

At current presentation, bronchoscopy was performed to evaluate the possibility of tracheal recurrence. No endobronchial lesions were observed, and given the absence of clinical or radiological indicators of tracheal disease, further biopsy of the trachea was not deemed necessary. Instead, direct laryngomicroscopy with biopsy was performed due to the presence of a subglottic mass. Histopathological examination of two separate biopsy specimens, measuring approximately 3 and 4 mm in greatest dimension, confirmed the diagnosis of LACC.

Definitive surgical treatment included total laryngectomy, bilateral selective neck dissection, and right hemithyroidectomy. Intraoperatively, the tumor involved both true vocal cords, the anterior commissure, and extended into the subglottic region.

Surgical exploration revealed tumor involvement of both true vocal cords and the anterior commissure, extending into the subglottic area. Histopathological evaluation of the surgical specimen confirmed ACC, staged as pT4aN0. On gross examination, a transglottic tumor measuring 44 × 30 × 15 mm was identified (Figure 1c). The radial resection margin was inked, and eight representative tissue blocks were submitted for microscopic examination, sampling the inferior tracheal ring, supraglottis, and the glottic and subglottic regions bilaterally. Microscopically, the tumor was composed of tubular and cribriform structures formed by luminal and abluminal cells (Figures 2A, 2B, and 2C). Vascular invasion was confirmed in routine sections; no perineural invasion was identified. No overt foci of necrosis were identified. Tumor infiltrated the thyroid cartilage. No adverse or unanticipated perioperative or post‐treatment events were observed during the reported follow‐up period. The case was reviewed by the institutional Malignant Diseases Advisory Board, which recommended close clinical and radiological follow‐up. At the 24‐month follow‐up, the patient had no signs of recurrence and maintained a stable clinical status (Table 1).

Figure 2Case no. 1: histopathological findings of laryngeal adenoid cystic carcinoma (H&E). (A) Low‐power view showed a submucosal tumor composed predominantly of tubular/gland‐like structures (arrows); the cribriform/tubular area is highlighted (dashed ellipse) within a hyalinized stromal background (∗). A dashed rectangle indicates the region digitally enlarged in panel c. Scale bar: 2000 *μ*m. (B) Higher magnification showed tumor infiltration into thyroid cartilage (arrows). Scale bar: 500 *μ*m. (C) Digitally enlarged detail from panel a highlighting basaloid tumor cells with hyperchromatic nuclei and scant cytoplasm lining tubular/gland‐like structures (arrows).

**Figure:**
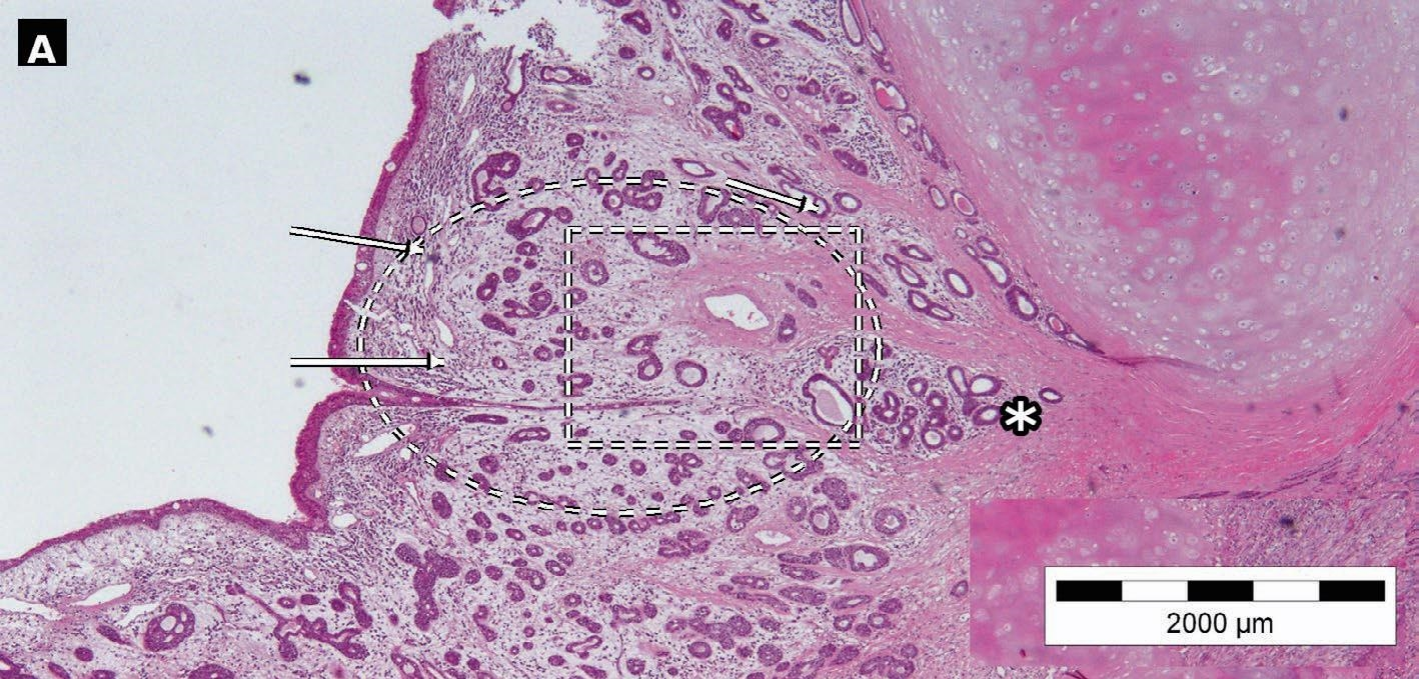

**Figure:**
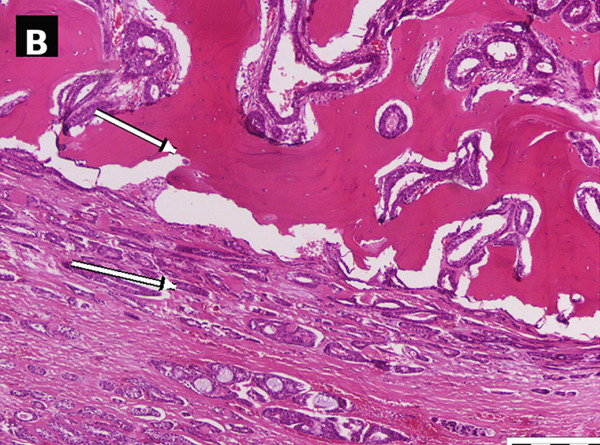

**Figure:**
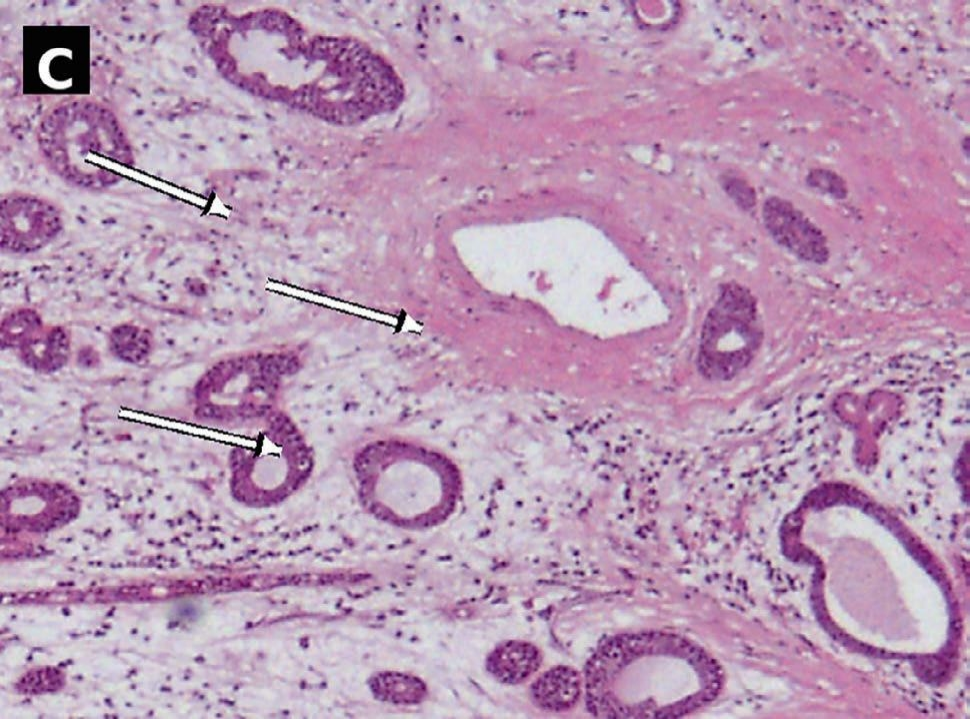

### 2.2. Case No. 2

**Figure 4:**
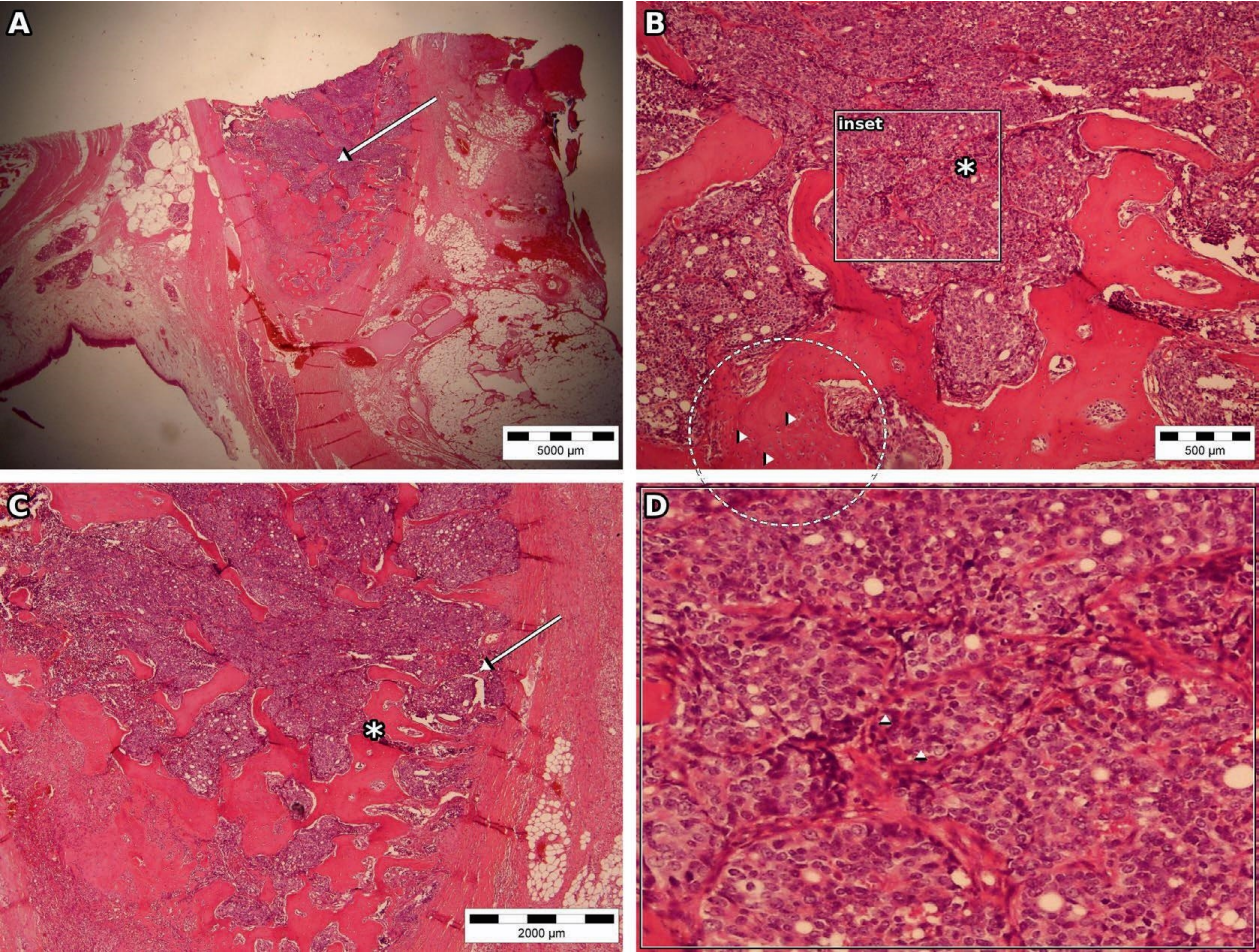
Case no. 2: histopathological findings of laryngeal adenoid cystic carcinoma (H&E). (A) Low‐power view showed tumor infiltration of the thyroid cartilage (arrow) (Scale bar: 5000 *μ*m). (B) Cribriform architecture with pseudocystic spaces (arrowheads) and stromal component (Scale bar: 500 *μ*m); boxed area corresponds to panel D. (C) Tumor nests invading adjacent soft tissues and approaching the perichondrium (arrow) with stromal component (Scale bar: 2000 *μ*m). (D) Digitally enlarged detail from the boxed region in (B) highlighting basaloid tumor cells and nuclear features (arrowheads).

A 79‐year‐old male patient presented with a history of persistent hoarseness lasting several months. Previously, the patient underwent two laryngomicroscopic examinations at another hospital and two incisional biopsies were performed. Despite two prior biopsies performed at another institution, histopathological evaluation at the time revealed no malignancy. The precise extent of tissue block sampling and sectioning was unavailable for review, but the negative findings could have been related to the tumor’s submucosal and focal growth pattern, which posed a challenge even in adequately collected samples. Clinical evaluation revealed right‐sided laryngeal immobility accompanied by right ventricular hypertrophy. A neck CT scan demonstrated a hyperdense lesion adjacent to the glottis, exerting pressure on the right vallecula and causing laryngeal stenosis (Figure 3A). Additionally, erosion of the thyroid cartilage was noted. No abnormalities were detected on the chest CT scan or abdominal ultrasound examination. Given the prior negative findings for malignancy, an open biopsy procedure was performed, and histopathological evaluation of the biopsy specimen revealed features consistent with ACC. Subsequently, the patient underwent total laryngectomy with selective right‐sided neck dissection. On gross examination, a predominantly right‐sided transglottic tumor measuring 26 × 21 × 13 mm was identified (Figure 3B). The radial resection margin was inked, and eight representative tissue blocks were submitted for microscopic examination, sampling the inferior tracheal ring, supraglottis, and the glottic and subglottic regions bilaterally. Microscopically, the tumor showed solid and tubular architecture, composed of basaloid cells and pseudoglandular structures lined by luminal and abluminal cells (Figure 4). Lymphovascular invasion was present, but no perineural invasion was identified. No overt foci of necrosis were identified. The tumor infiltrated the thyroid cartilage and was present 0.5 mm from the closest radial resection margin. Histopathological analysis confirmed the tumor as pT4aN0. Postoperative management included radiotherapy, delivering a total dose of 63 Gy in 30 fractions. During the reported follow‐up period, no adverse or unexpected perioperative or post‐treatment events were observed. Seventeen months after surgery, the patient remains in good health, with no evidence of recurrence (Table 2).

Figure 3Case no. 2: CT and intraoperative findings of laryngeal tumor. (A) Axial CT scan of the neck revealed a hyperdense paraglottic mass, resulting in compression of the right vallecula and laryngeal stenosis at the level of the glottis extending towards the caudal region (arrow). (B) Tumor mass of the right larynx infiltrating cricoid cartilage measuring 26 × 21 × 13 mm (arrow).

**Figure:**
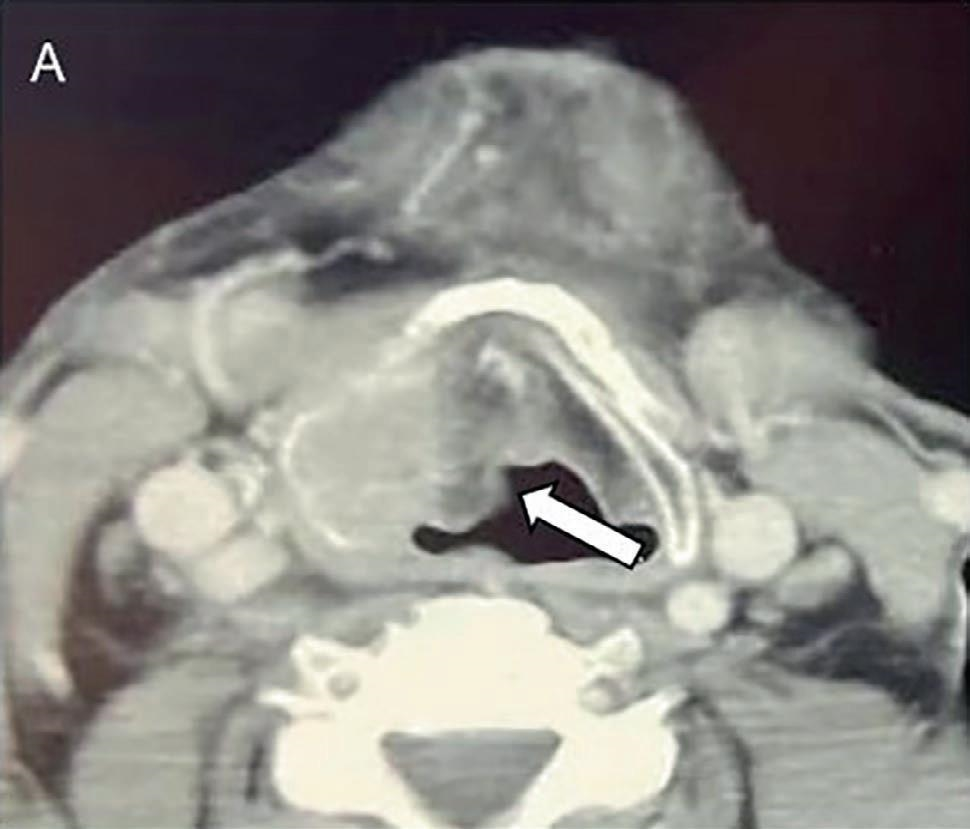

**Figure:**
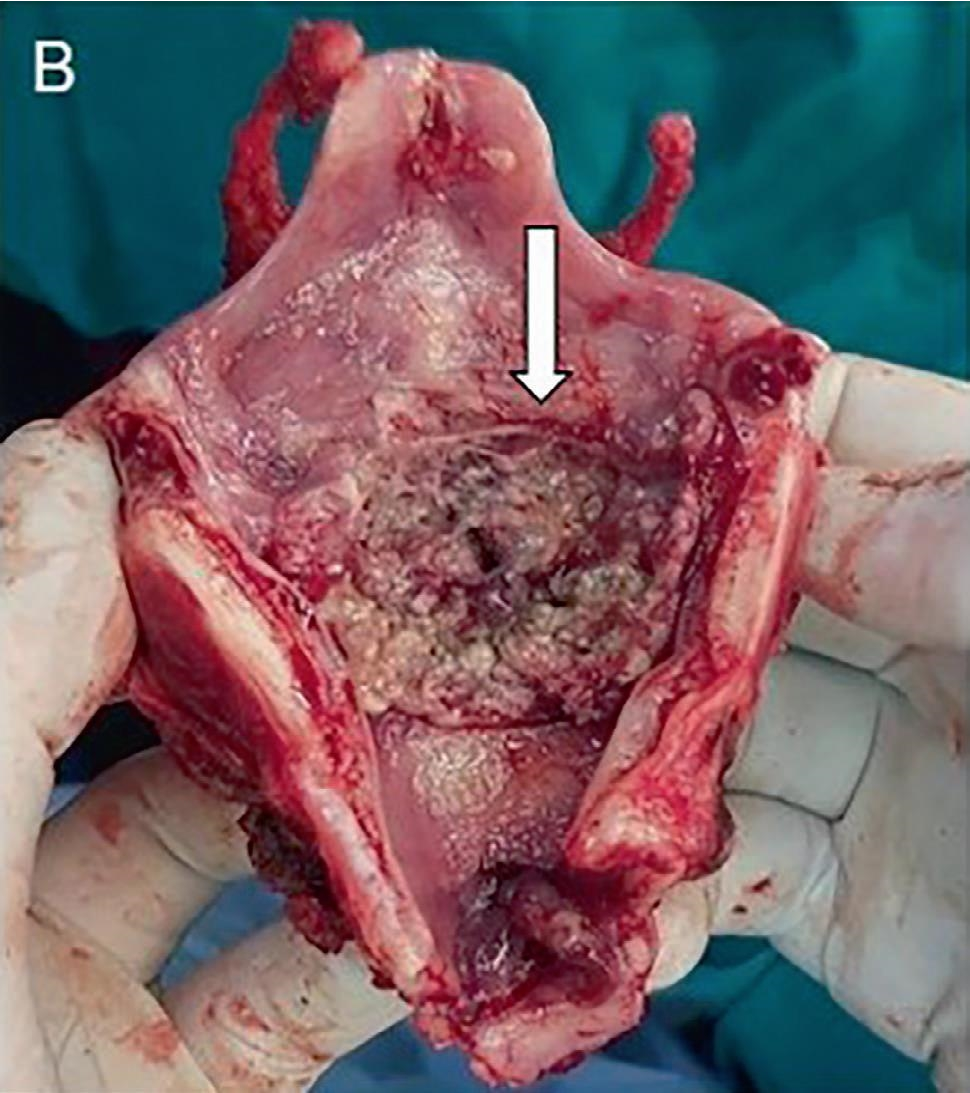

**Table 1:** Case no. 1. Timeline of historical and current information from the episode of care.

Time point	Clinical course/diagnostic findings	Management/outcome
9 years before current presentation	Tracheal adenoid cystic carcinoma was diagnosed.	Systemic chemotherapy with gemcitabine/cisplatin was initiated.
Initial treatment period	Follow‐up imaging showed multiple liver and adrenal lesions considered radiologically suspicious for metastases; histopathologic confirmation was not obtained.	Six additional cycles of gemcitabine/cisplatin were administered, followed by maintenance gemcitabine.
During serial imaging follow‐up	Previously described liver and adrenal lesions were no longer detected; only minimal non‐progressive residual hepatic hypodensities remained.	Findings were interpreted as nonspecific and without radiologic evidence of active metastatic disease.
2 years after initial therapy	Disease recurrence was treated as locoregional recurrence of the prior tracheal ACC.	External beam radiotherapy was delivered (44 Gy in 22 fractions), followed by complete radiological remission on CT.
5 months before current presentation	Persistent cough, fatigue, and progressive dysphagia developed.	Patient sought renewed otorhinolaryngologic evaluation.
Current presentation	Indirect laryngoscopy showed bilateral vocal fold paralysis, suspicious mucosal irregularity, and marked narrowing of the glottic airway. Neck CT revealed a right‐sided subglottic mass without cervical lymphadenopathy. Chest CT and abdominal ultrasound showed no distant metastatic disease.	Bronchoscopy was performed to exclude tracheal recurrence; no endobronchial lesion was found.
Diagnostic confirmation	Direct laryngomicroscopy with biopsy of the subglottic lesion was performed; two biopsy fragments (approximately 3 and 4 mm) confirmed laryngeal adenoid cystic carcinoma.	Definitive surgery was planned.
Definitive treatment	Intraoperative findings showed tumor involvement of both true vocal cords, the anterior commissure, and the subglottic region. Grossly, the transglottic tumor measured 44 × 30 × 15 mm.	Total laryngectomy, bilateral selective neck dissection, and right hemithyroidectomy were performed.
Postoperative pathology/board review	Histopathology confirmed adenoid cystic carcinoma, pT4aN0, with tubular and cribriform architecture, vascular invasion, and thyroid cartilage infiltration; no perineural invasion or necrosis was identified.	Case was reviewed by the institutional Malignant Diseases Advisory Board, which recommended close clinical and radiological follow‐up.
24 months after surgery	No signs of recurrence; clinical status remained stable.	Continued surveillance.

**Table 2:** Case no 2. Timeline of historical and current information from the episode of care.

Time point	Clinical course/diagnostic findings	Management/outcome
Several months before presentation	Persistent hoarseness developed and lasted for several months.	Patient was evaluated at another hospital.
Before referral	Two laryngomicroscopic examinations and two incisional biopsies were performed at another institution; histopathology did not reveal malignancy.	Because of persistent clinical suspicion, further evaluation was continued.
Current presentation	Clinical examination showed right‐sided laryngeal immobility and right ventricular fold hypertrophy. Neck CT demonstrated a hyperdense paraglottic/glottic lesion with compression of the right vallecula, laryngeal stenosis, and thyroid cartilage erosion. Chest CT and abdominal ultrasound showed no distant disease.	Open biopsy was selected because prior endoscopic biopsies were non‐diagnostic.
Diagnostic confirmation	Open biopsy specimen showed features consistent with adenoid cystic carcinoma.	Definitive surgical treatment was planned.
Definitive treatment	Predominantly right‐sided transglottic tumor was identified intraoperatively (26 × 21 × 13 mm).	Total laryngectomy with selective right‐sided neck dissection was performed.
Postoperative pathology	Histopathology confirmed adenoid cystic carcinoma, pT4aN0, with solid and tubular architecture, lymphovascular invasion, thyroid cartilage infiltration, and a closest radial margin of 0.5 mm.	Adjuvant radiotherapy was indicated.
Postoperative adjuvant therapy	Radiotherapy was delivered after surgery.	Total dose 63 Gy in 30 fractions.
17 months after surgery	No evidence of recurrence; patient remained in good general condition.	Ongoing surveillance recommended.

**Table 3:** Differential diagnosis of submucosal laryngeal tumors: key clinico‐pathologic features.

Feature	Laryngeal adenoid cystic carcinoma (LACC)	Mucoepidermoid carcinoma (MEC)	Neuroendocrine carcinoma (NEC∗)
Typical growth/biopsy pitfall	Submucosal, infiltrative; superficial biopsies can miss diagnostic areas	Can be submucosal; may be cystic; small biopsies may undergrade	Often submucosal/deep infiltrative; crush artifact in small biopsies
Histology (hallmarks)	Cribriform/tubular ± solid pattern; hyalinized stroma; frequent perineural tendency (variable)	Mixture of mucous + intermediate + squamoid cells; cystic spaces; mucin	Organoid nests/trabeculae/rosettes (well‐differentiated) or sheets of small/large cells with necrosis (poorly‐differentiated)
Cell/nuclear features	Basaloid cells with hyperchromatic nuclei, scant cytoplasm; dual cell population (luminal/abluminal)	Variable; mucous cells with intracytoplasmic mucin; intermediate cells common	“Salt‐and‐pepper” chromatin (well‐ differentiated) or marked atypia + high mitoses (poorly‐differentiated)
Stroma	Hyalinized (“hyaline‐rich”, basement membrane‐like)	Usually no classic hyaline stroma; may show fibrosis	Variable; often minimal stroma in high‐grade NEC
Mucin	Typically absent or minimal true mucin	Present (mucicarmine/PAS‐D positive)	Typically negative
IHC (practical panel)	CK7/AE1‐AE3; CD117 (c‐KIT); SOX10/S100; myoepithelial markers (p63/p40, SMA/calponin) in abluminal cells	CK7/AE1‐AE3; p63; mucin stains; (S100 variable); may lack a distinct myoepithelial (abluminal) cell layer.	Synaptophysin, chromogranin, INSM1; Ki‐67 (grading); cytokeratins often + in NEC
Molecular alterations (helpful, if available)	MYB pathway alterations (e.g., MYB–NFIB) in many ACCs	CRTC1/3–MAML2 fusion in many MECs	No single defining fusion; classification rests on morphology + NE markers + grade
Clinical behavior	Indolent but locally aggressive; late recurrences and distant metastases possible	Prognosis grade‐dependent (low vs high grade)	Aggressive in poorly differentiated NEC; early metastases common
Treatment (typical)	Surgery ± adjuvant RT; neck dissection generally not routine unless nodes	Surgery ± RT; approach depends on grade/stage	Multimodal; often chemo‐RT in high‐grade NEC + staging for systemic disease

∗NEC includes well‐differentiated neuroendocrine tumor and poorly differentiated neuroendocrine carcinoma (small/large cell); behavior and Ki‐67 differ substantially.

## 3. Discussion

From a histopathological perspective, the majority (90%–95%) of laryngeal cancer cases are diagnosed as squamous cell carcinoma, although other pathohistological types can also manifest [1]. Among the rarest forms is ACC, previously referred to as cylindroma [2]. The term cylindroma was in usage until 1953 when it was renamed ACC [2]. LACC represents less than 1% of all laryngeal tumors and typically originates from minor salivary glands, which are sparsely distributed in the laryngeal and tracheal mucosa [3]. The subglottis serves as the most common site for this tumor (58.2%) [6].

These tumors frequently manifest as submucosal masses at the site of salivary glands. As a result, these tumors often pose specific challenges for biopsy procedures due to their submucosal growth pattern. Superficial biopsy fragments may be insufficient for diagnosis, especially when small in size or limited to overlying mucosa. There is also a risk of sampling error, as the infiltrative components may lie deeper within the laryngeal tissue and be easily missed during routine endoscopic biopsies. In the case described here (case no. 2), diagnosis was not achieved until laryngofissure and open biopsy were performed.

Given the submucosal and often deeply infiltrative growth pattern of LACC, limited or superficial biopsies may fail to capture diagnostic areas or may be misinterpreted as benign/inflammatory changes. In this setting, the main differential diagnoses include other submucosal laryngeal malignancies, most notably mucoepidermoid carcinoma and neuroendocrine tumors, which can share overlapping clinical or architectural features on small specimens. To facilitate diagnostic orientation, Table 3 summarizes practical clinico‐pathologic clues, immunohistochemical markers, and key molecular features that help distinguish LACC from these entities.

The etiopathogenesis of LACC remains incompletely understood and is likely multifactorial; no consistent, established risk factors have been identified so far [3]. As per certain studies, malignant salivary gland tumors, such as LACC, are considered to develop as a result of genomic alterations [7]. The most prevalent among these alterations is the chromosomal translocation t(6;9), or less frequently t(8;9), resulting in fusion of the MYB or MYBL1 oncogene with the NFIB transcription factor gene [7]. More frequently, the disease manifests between the ages of 50 and 60; nevertheless, both of these patients were older than 70 years [6]. Clinically, ACC is characterized by slow‐growing, painless lesions [4]. The symptoms of LACC vary based on its location and size.

Diagnosis of LACC is frequently delayed due to its submucosal growth pattern and histological overlap with benign or inflammatory lesions. In our second case, two previous incisional biopsies failed to reveal malignancy. It was unclear whether the tissue blocks were entirely evaluated or if diagnostic areas were missed due to the focal and deeply infiltrative nature of the tumor. In addition, scar tissue following the first biopsy may have obscured critical stromal features in subsequent specimens, contributing to further diagnostic uncertainty. This underscored the importance of adequate deep tissue sampling and, when clinical suspicion persists, close clinicopathological correlation and careful re‐evaluation of available material, with repeat or open surgical biopsy when necessary. Involvement of regional lymph nodes is observed in about 10%–15% of cases, with more frequent distant metastases (35%–50%), primarily affecting the lungs, followed by the bones and liver [3]. Distant metastases can emerge years following the initial diagnosis of the primary tumor. Hence, long‐term follow‐up is essential for these patients [4]. In our first case, the patient had a history of tracheal ACC treated 9 years earlier. The current finding of a subglottic laryngeal tumor raised the question of whether this represented a late distant metastasis from the previously treated tracheal ACC or a new primary (de novo) laryngeal ACC. Both possibilities are exceedingly rare, and a definitive distinction between metastatic disease and a second primary tumor could not be made without molecular comparison [8]. Several clinical and radiological factors favored a second primary tumor: the long interval between the two clinical events (over 9 years), the absence of direct continuity with the original tracheal site. Although metastatic disease was considered, imaging procedures did not demonstrate additional metastatic lesions, and histopathological evaluation did not provide evidence to confirm metastatic spread. Moreover, the subglottic location of the new mass, without radiological or bronchoscopic evidence of tracheal involvement, supported the hypothesis of a de novo laryngeal primary origin. Histopathological examination showed a cribriform/tubular growth pattern consistent with ACC but did not provide features that could establish clonal relatedness. Late distant metastases are a recognized clinical characteristic of ACC, and the larynx—particularly the subglottic region—may rarely be involved. Molecular testing (e.g., comparative analysis for MYB–NFIB fusion or other clonality approaches) could help determine whether the tumors share a common origin; however, such testing was not available/performed. Given the absence of evidence for metastatic spread or direct extension and the isolated nature of the lesion, the tumor was managed as a primary LACC.

Considering its rarity, there are currently no clearly defined treatment guidelines. Because of the relative radioresistance of this tumor type, surgical resection followed by postoperative radiotherapy is advised to reduce the risk of recurrence [9]. Because regional nodal metastasis appears uncommon in LACC, elective neck dissection is not routinely performed in clinically node‐negative patients. However, performing a neck dissection is justified in cases where regional lymph node metastases are detected by clinical examination or imaging procedures [10]. Even though no pathological lymph nodes were detected on clinical examination or imaging procedures, neck dissection was still performed in both cases, taking into account tumor size, patient age, and overall health status. The five‐year survival rate following surgical resection for patients with LACC typically falls within the range of 12‐17% [11]. As of 24 and 17 months post‐treatment, these patients remain recurrence‐free. Given the diversity of histological patterns and their possible prognostic implications, future multicenter studies could help determine whether more refined histological classification of LACC might inform treatment decisions and follow‐up strategies. Until then, complete surgical excision with individualized use of adjuvant therapy remains the cornerstone of management.

## 4. Conclusion

LACC is an uncommon malignancy with indolent yet infiltrative growth. In both cases presented, the tumors demonstrated advanced local spread with cartilage invasion and transglottic extension, highlighting the importance of thorough histopathological assessment in guiding treatment. The first case displayed a cribriform/tubular growth pattern with deep infiltration into the thyroid cartilage but no perineural invasion or necrosis, which supported the decision for total laryngectomy with bilateral neck dissection. In contrast, the second case showed a predominantly solid pattern with close resection margins and prompted the use of adjuvant radiotherapy following surgery. These pathology‐driven decisions underscore the need to integrate microscopic features—such as growth pattern, margin status, and cartilage involvement—into individualized treatment planning. The differences in histological architecture between the two cases, despite the same tumor entity, further emphasize the heterogeneity of LACC and the necessity of precise pathological characterization. Ultimately, these two cases illustrate the diagnostic and therapeutic challenges of LACC, particularly in elderly patients without classical risk factors. Long‐term follow‐up remains essential due to the potential for late recurrence and distant spread of the tumor. The findings support the value of detailed histopathological evaluation not only for accurate diagnosis but also for tailoring appropriate and effective treatment strategies.

## Author Contributions

Katarina Jovanovic: contributed to the project creation, conceptualization, methodology, data collection, and writing and editing.

Sasa Jakovljevic: contributed to the conceptualization, guidance, methodology and writing – review and editing.

Neda Mladenovic: contributed to data collection and writing – review and editing.

Nemanja Radivojevic: contributed to data collection and writing – review and editing.

Djurdjina Kablar: contributed to data collection.

Ana Marija Tomic: contributed to data collection.

Hristina Glogovac: contributed to data collection, and writing – review and editing.

Zoran Dudvarski: contributed to the supervision, conceptualization, methodology, guidance, and writing – review and editing.

## Funding

The authors received no specific funding for this work.

## Ethics Statement

The authors are accountable for all aspects of the work in ensuring that questions related to the accuracy or integrity of any part of the work are appropriately investigated and resolved. The study was conducted in accordance with the Declaration of Helsinki (as revised in 2013). Informed consent was obtained. Consent to publish was obtained.

## Conflicts of Interest

The authors declare no conflicts of interest.

## Supporting information


**Supporting Information** Additional supporting information can be found online in the Supporting Information section.

## Data Availability

The data that support the findings of this study are available from the corresponding author upon reasonable request.
