# Supplementary material for: Laryngeal Adenoid Cystic Carcinoma—Two Case Reports and Literature Review
Source: Case Rep Pathol. 2026 Apr 8;2026:4744129. doi: 10.1155/crip/4744129 (PMC13058926; doi:10.1155/crip/4744129)
Supplement: Supplementary file 1 — Supporting Information Additional supporting information can be found online in the Supporting Information section. [file CRIP-2026-4744129-s001.docx]

**Table 1. Differential diagnosis of submucosal laryngeal tumors: key clinico-pathologic features**

| **Feature** | **Laryngeal adenoid cystic carcinoma (LACC)** | **Mucoepidermoid carcinoma (MEC)** | **Neuroendocrine carcinoma (NEC*)** |
| --- | --- | --- | --- |
| Typical growth / biopsy pitfall | Submucosal, infiltrative; superficial biopsies can miss diagnostic areas | Can be submucosal; may be cystic; small biopsies may undergrade | Often submucosal/deep infiltrative; crush artifact in small biopsies |
| Histology (hallmarks) | Cribriform/tubular ± solid pattern; hyalinized stroma; frequent perineural tendency (var.) | Mixture of mucous + intermediate + squamoid cells; cystic spaces; mucin | Organoid nests/trabeculae/rosettes (well-diff) or sheets of small/large cells with necrosis (poorly diff) |
| Cell/nuclear features | Basaloid cells with hyperchromatic nuclei, scant cytoplasm; dual cell population (luminal/abluminal) | Variable; mucous cells with intracytoplasmic mucin; intermediate cells common | “Salt-and-pepper” chromatin (well-diff) or marked atypia + high mitoses (poorly diff) |
| Stroma | Hyalinized (“hyaline-rich”, basement membrane-like) | Usually no classic hyaline stroma; may show fibrosis | Variable; often minimal stroma in high-grade NEC |
| Mucin | Typically absent or minimal true mucin | Present (mucicarmine/PAS-D positive) | Usually negative |
| IHC (practical panel) | CK7/AE1-AE3; CD117 (c-KIT); SOX10/S100; myoepithelial markers (p63/p40, SMA/calponin) in abluminal cells | CK7/AE1-AE3; p63; mucin stains; (S100 variable); may lack myoepithelial dual layer | Synaptophysin, chromogranin, INSM1; Ki-67 (grading); cytokeratins often + in NEC |
| Molecular (helpful, if available) | MYB pathway alterations (e.g., MYB–NFIB) in many ACCs | CRTC1/3–MAML2 fusion in many MECs | No single defining fusion; classification rests on morphology + NE markers + grade |
| Clinical behavior | Indolent but locally aggressive; late recurrences and distant metastases possible | Prognosis grade-dependent (low vs high grade) | Aggressive in poorly differentiated NEC; early metastases common |
| Treatment (typical) | Surgery ± adjuvant RT; neck dissection generally not routine unless nodes | Surgery ± RT; approach depends on grade/stage | Multimodal; often chemo-RT in high-grade NEC + staging for systemic disease |

**NEC includes well-differentiated neuroendocrine tumor and poorly differentiated neuroendocrine carcinoma (small/large cell); behavior and Ki-67 differ substantially.*
